# Natural Selection Towards Wild-Type in Composite Cross Populations of Winter Wheat

**DOI:** 10.3389/fpls.2019.01757

**Published:** 2020-02-25

**Authors:** Samuel Knapp, Thomas F. Döring, Hannah E. Jones, John Snape, Luzie U. Wingen, Martin S. Wolfe, Michelle Leverington-Waite, Simon Griffiths

**Affiliations:** ^1^ John Innes Centre, Norwich Research Park, Norwich, United Kingdom; ^2^ Plant Nutrition, Technical University of Munich, Freising, Germany; ^3^ The Organic Research Centre, Hamstead Marshall, United Kingdom; ^4^ Agroecology and Organic Farming Group, University of Bonn, Bonn, Germany; ^5^ The School of Agriculture, Policy and Development, University of Reading, Reading, United Kingdom

**Keywords:** cropping system, evolution, genetic diversity, natural selection, plant height

## Abstract

Most of our crops are grown in monoculture with single genotypes grown over wide acreage. An alternative approach, where segregating populations are used as crops, is an exciting possibility, but outcomes of natural selection upon this type of crop are not well understood. We tracked allelic frequency changes in evolving composite cross populations of wheat grown over 10 generations under organic and conventional farming. At three generations, each population was genotyped with 19 SSR and 8 SNP markers. The latter were diagnostic for major functional genes. Gene diversity was constant at SSR markers but decreased over time for SNP markers. Population differentiation between the four locations could not be detected, suggesting that organic *vs.* non-organic crop management did not drive allele frequency changes. However, we did see changes for genes controlling plant height and phenology in all populations independently and consistently. We interpret these changes as the result of a consistent natural selection towards wild-type. Independent selection for alleles that are associated with plant height suggests that competition for light was central, resulting in the predominance of stronger intraspecific competitors, and highlighting a potential trade-off between individual and population performance.

## Introduction

Successful crop production depends on varieties that are well adapted to a target environment (Cooper and Hammer, 1996; Atlin et al., 2017) but sufficiently widely adapted so that breeding and seed production is economically viable. As a result, a large proportion of the harvested area is occupied by a few major inbreeding crops (e.g. wheat, barley, rice) and within any one farm, large blocks of each crop comprise single genotypes. This bears risks of vulnerability to diseases (Brown and Hovmøller, 2002) and limited adaptability to local conditions (Mercer and Perales, 2010).

A potential response to these drawbacks is the use of genetically diverse populations instead of clonal crops (Litrico and Violle, 2015). Crop populations can be created by mixing different varieties (Finckh and Wolfe, 2006) or by inter-crossing varieties and mixing the progenies (Suneson, 1956), which, in combination with harvesting and re-sowing each generation, is called evolutionary plant breeding (Suneson, 1956; Döring et al., 2011). Genotypes better adapted to local conditions should have more progeny and thus increase in frequency and over time could result in better locally adapted genotypes and increased grain yield (Döring et al., 2011).

Early wheat studies describe yield increases, with reported rates of genetic gain comparable to those of mainstream breeding (Suneson, 1956). Similar results were reported by Allard (1988) and for biotic stress, Le Boulc’h et al. (1994) found increased resistance to powdery mildew as did Paillard et al. (2000). Furthermore, it was found that diverse winter wheat populations across France showed a differentiation in phenological development (Rhoné et al., 2010). Populations grown over several generations in Northern France with colder winters flowered later than populations grown in Southern France with risk of drought at the end of the growing season. Recently, Bertholdsson et al. (2016) showed that seedling traits of winter wheat composite cross populations (CCPs) were differentially selected in organic *vs.* conventional management systems, with the populations maintained under organic management showing an increase in seedling root length and root weight, while populations under conventional management showed no such increase. This suggests that the selection CCPs are subjected to can lead to adaptation to locally prevailing conditions and management systems.

These positive results were achieved in spite of the trade-off between individual plant fitness and population performance (Weiner et al., 2010; Denison, 2012; Anten and Vermeulen, 2016). Natural selection acts on individuals but population performance is the central variable in crop production. Individual fitness in a population strongly depends on competition-related traits such as plant height, but investment by individual plants in competition may reduce their potential for grain yield (Weiner et al., 2010). Accordingly, harvest index in cereals, i.e. the proportion of grain yield in total biomass, decreases with increasing intra-specific competition among crop plants with increasing density (Weiner and Freckleton, 2010). However, under no-herbicide conditions of organic farming, where weeds are often more abundant than in conventional cropping systems (Gabriel et al., 2013), the same competition-related traits may be of advantage.

At the genetic level, a small number of major genes have been deployed in modern breeding. Gain of function mutations and alien introgressions that are absent in wild wheat, possessing the wild-type alleles, now occur at high allele frequency in elite bread wheat. These include *Rht-1* (Peng et al., 1999), *Ppd-1* (Beales et al., 2007), and the *1B/1R* rye introgression (Schlegel and Korzun, 1997). It is not well understood how these mutations, selected in the 20th century under monoculture agronomy, and their corresponding wild-type alleles, might influence the overall performance of a segregating population and respond to natural selection.

Our first objective was to find out whether selection can lead to genetic differentiation reflecting adaptation to different management conditions, and if the signature of this selection can be detected for a set of genes with particular importance for competition. Secondly, we investigated if the observed selection of certain alleles can be explained through their phenotypic effect on plant height, heading date, yield, and yield components in individual plants within the population (mixed stand). Thirdly, to investigate possible trade-offs between individual and population performance we compared these phenotypic effects within individual plants and in pure stands. We conducted this investigation on CCPs of winter wheat grown with minimal artificial selection for 10 generations in four locations, two organically and two conventionally managed, in Southern England.

Besides advancing the basic understanding of evolutionary principles, this study has important implications for the deployment of diversity in crop production. If natural selection is counteracting population performance, natural selection will not produce crop populations that are superior in yield, quality, or other agronomic traits. This understanding is crucial for the role and design of plant breeding. However, if the direction of selection occurs predictably it may be possible to pre-select populations using marker-assisted selection, so that fewer generations of on-farm selection are required. Furthermore, alleles that would increase in frequency, but be detrimental to crop performance, could be removed before allowing for natural selection.

## Material and Methods

### Creation of Populations and Description of Locations

**Figure 1:**
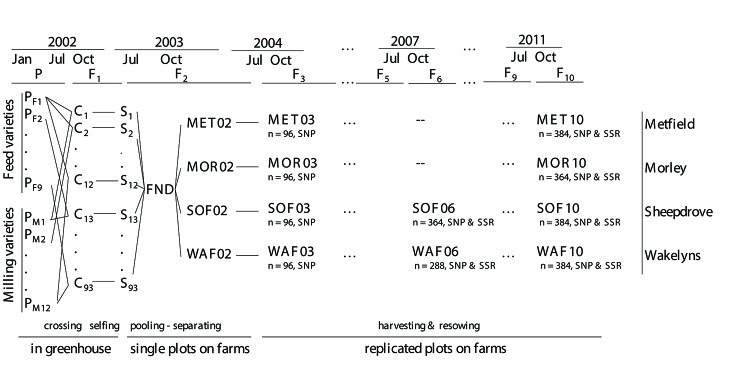
Schematic overview of the crossing scheme of the composite cross populations (CCP) and of the sampled populations (bold fonts). The number of sampled individuals (n) and the sets of markers that were analyzed are shown below each population.

The CCPs were created by inter-crossing two sets of wheat varieties: eight feed varieties and 11 milling varieties, plus the variety Bezostaya which was in both sets (**Figure 1** and **Table S1**). Nineteen of the 20 varieties were chosen for a previous field assessment with the aim to represent successful varieties or parents of successful varieties in Western Europe and to obtain a high level of genetic diversity (Jones et al., 2010). They differed in major phenotypic characteristics like plant height, earliness, vernalization requirement, and baking quality. F_1_ plants were self-fertilized, the number of F_2_ seeds from each cross was recorded, and all seeds from each cross were subsequently pooled, i.e. not in equal proportion. Seeds from 93 successful crosses (mean of 957 seeds per cross and range: 37 to 2,569), entered the pooled founding population, subsequently termed FND. The pooled seeds were separated and sown by hand at each of the four locations in October 2003 in single plots (**Figure 1** and see also Döring et al., 2015). In subsequent generations, the populations were grown in a randomized complete block design (RCBD) with other varieties and populations with three replications (see Döring et al. (2015)). The average plot size was 25 m^2^ and the target plant density 425 plants/m^2^, giving an average demographic populations size of around 32,000 plants. Each year the seeds from all three replicates within a location were pooled, and a proportion was sampled for resowing the next generation without any artificial selection. Until the last sampling of seeds for this study, populations evolved for 10 generations.

CCPs were grown at four locations: two organically managed: Wakelyns Agroforestry (WAF) in Suffolk (52° 39′N, 1° 17′E) and Sheepdrove Organic Farm (SOF) in Berkshire (51° 31′N, 1° 30′W); and two conventionally managed: Metfield Hall Farm (MET), directly adjacent to WAF in Suffolk (52° 41′N, 1° 29′E) and Morley Research Station (MOR) in Norfolk (52° 56′N, 1° 10′E). Fertilizer, pesticide, and growth regulator applications at MET and MOR followed commercial practice. At the organic locations, WAF and SOF, no pesticides were applied and weeds were controlled mechanically and through rotational design. As the experiments were integrated in the local crop rotation, the trials were moved within the crop rotation at each location. Therefore, the preceding crop remained constant over the course of the experiment (legume–grass mixtures at the organic locations, winter wheat at MET, and winter rape seed at MOR). For detailed descriptions of climatic and soil conditions see Jones et al. (2010) and Döring et al. (2015).

### Sampling of Plant Material and Phenotyping

At the organic locations, SOF and WAF, in generation 6 and 10, a total of 501 individual plants in equal proportions from each of the three replicate plots were tagged in the field, and plant height and heading date (day when ear was half-way emerged from flag leaf) were recorded. Sampling of individual plants was carried out only at the organic locations due to the proximity of these locations to the research infrastructure. Individual plants were harvested, threshed, and grain yield of the whole plant was determined. From the plants that had produced seeds, plants for DNA extraction were sampled randomly (see **Figure 1** for the final numbers of plants). From these plants three random seeds were germinated and leaf tissue from one seedling was sampled for DNA extraction. At generation 3, from each location 150 seeds, and at generation 10 from MET and MOR, 500 seeds were sampled from pooled plot harvests for genotyping, germinated, and DNA was extracted from leaf tissue of the seedlings (final sample number given in **Figure 1**). As the genotype of the parental lines was crucial for the generation of the virtual FND population (see below), DNA was extracted from five different seedlings per parental variety.

To evaluate the effect of the markers on pure stand performance, common plot trials with 19 of the total 20 parental varieties (except Norman) were assessed at the same four locations for plant height, obtained from 10 randomly chosen plants per plot, grain yield, and yield components. These trials were conducted in three consecutive years (2005–2007) in a RCBD design with three replicates. For more detailed descriptions see Jones et al. (2010).

### Genotyping

After running a set of 70 publicly available SSR markers on the parental varieties and assessing the number of alleles and amplification quality, a subset of 18 SSR markers was chosen: 15 markers from Röder et al. (1998) (*gwm44*, *gwm46*, *gwm165*, *gwm186*, *gwm190*, *gwm213*, *gwm234*, *gwm325*, *gwm337*, *gwm469*, *gwm539*, *gwm583*, *gwm610*, and *gwm626*, of which *gwm44* and *gwm165* produced two loci), two markers from Stephenson et al. (1998) (*psp3100* and *psp3103*) and each one marker from Edwards et al. (1996) (*wmc56*) and Somers et al. (2004) (*barc134*). As two markers produced two loci, all-in-all 20 SSR loci were used for the genotype analysis.

Additionally, eight SNP markers were included, which were shown to be diagnostic for major genes involved in plant height (*Rht-B1* and *Rht-D1*), vernalization requirement (*Vrn-A1*), photoperiod response (*Ppd-A1*, *Ppd-B1*, *Ppd-D1*, and *Ppd-D1*(*D2*)), and one marker linked to the *1B/1R* chromosome translocation from rye (Schlegel and Korzun, 1997). Information on the SNP markers can be found at http://www.cerealsdb.uk.net/cerealgenomics/CerealsDB/kasp_download.php.

### Statistical Analysis

#### Creation of a Virtual Founding Population

As no seeds were kept from the original pooled founding population, the genotypic composition of this population could not be determined directly. Instead, we generated a virtual founding population (FND) by creating the heterozygous genotypes of each cross, based on the genotype of the parental varieties. Subsequently we added the genotypes of each cross to the FND containing in total 10,000 individuals, proportionally to the recorded number of seeds that went into the “real” founding population (see above). The final number of the genotype from the cross of line i and line j in the FND is thus $n_{ij}=\frac{s_{ij}}{\sum^{}s_{ij}}*10,000,$ where *s_ij_* is the number of seeds from the cross of line i and line j, ∑*s*_*ij*_ is the sum of seeds from all crosses, and *n_ij_* was rounded to the nearest integer. We compared this approach to using the weight instead of the number of seeds (*s_ij_* as the weight of seeds) and to mixing parental genotypes instead of the genotypes of the crosses proportionally $(n_{i}=\frac{s_{i}}{\sum^{}s_{i}}*10,000),$ where *s_i_* is the number of seeds of each parental variety. The approach based on the weight of the seeds had a very small impact on the resulting allele frequencies with a mean absolute difference of 0.005. The latter approach of mixing parental genotypes proportionally showed no difference in allele frequencies. For this reason, we created the FND based on the number of seeds of each cross.

#### Treatment of SSR Markers

At the SSR loci, alleles that were absent in parental genotypes (due to mutation or migration) were removed from the dataset, as the focus was on the assessment of allele frequencies. Mutations and migrations were considered as random and thus assumed to have no biased effect on allele frequencies. To avoid further assumptions in the mathematical treatment of multi-allelic markers (Goldringer and Bataillon, 2004; Meirmans and Hedrick, 2010) and to be able to relate changes in allele frequencies to phenotypic effects of the alleles by reducing effects to only one allele, the marker data of the SSR markers were changed to bi-allelic markers. For each locus, the most frequent allele in FND was set as the first and all other alleles were pooled into the second allele. The number of alleles and parental genotypes carrying the most frequent allele are shown in **Table S1**.

#### Gene Diversity

As a measure of genetic diversity within populations, we estimated Nei’s gene diversity (*H_e_*), which equals the expected heterozygosity under Hardy–Weinberg equilibrium (Nei, 1973). We calculated *H_e_* for each locus and subsequently averaged over loci. Ninety-five percent confidence intervals (CIs) were generated by bootstrapping over loci with 5,000 bootstraps, thereby avoiding specific assumptions about the distribution of the estimated parameters. Gene diversity was calculated with the R-package *hierfstat* (Goudet, 2005).

#### Effective Population Size and Genetic Differentiation

Assessment of changes in allele frequencies requires an understanding of genetic drift, which is the random change of allele frequencies resulting from sampling of gametes in finite populations (Hedrick, 2005). Genetic drift depends on the effective population size (*N_e_*), which is the number of outcrossing individuals in an ideal Wright–Fisher population undergoing the same rate of genetic change (Wright, 1969). As pure genetic drift can also result in genetic differentiation, genetic differentiation is in turn related to the level of expected heterozygosity (Beaumont and Nichols, 1996; Excoffier et al., 2009a). Using data from all marker loci from generation 10, we employed the function to detect loci under selection in the *Arlequin* package (Excoffier et al., 2009b), to simulate the expected *F_ST_* null distribution under genetic drift, dependent on the expected heterozygosity with 20,000 simulations with 100 demes. The remaining loci were assumed to be neutral regarding differential selection. *N_e_* was then calculated from observed *F_ST_*, based on Hedrick (2005, p. 502) and the modification, that *F_ST_* is sampled from pairs of populations (see **Supplementary Method M1**):

$$N_{e}=t/\left(4\ln\left(-\frac{1}{F_{ST}-1}\right)\right).$$

*F_ST_* was estimated by Weir and Cockerham’s *F_ST_* (Weir and Cockerham, 1984) as implemented in *hierfstat*. As *F_ST_* was estimated in generation 10, and populations were separated in generation 2 (see **Figure 1**), we used *t* = 8. 95% CIs for N_e_ were calculated using CIs for *F_ST_* from the *boot.vc* function in *hierfstat.*

To compare observed versus expected changes in allele frequency under genetic drift, we calculated the 95% CIs of the allele frequency for each locus after *t* generations of genetic drift (Waples, 1989) as

$$95\%\;CI=p_{FND}\pm 1.96*\sqrt{p_{FND}*\left(1-p_{FND}\right)*\left[1-{\left(1-\frac{1}{2N_{e}}\right)}^{t}\right]},$$

where *p_FND_* is the allele frequency of the frequent allele in FND and *N_e_* the effective population size. Significance of changes was then assessed visually, if observed allele frequencies were smaller or greater than the CI expected under drift. We furthermore compared this method to the temporal method as first proposed by Waples (1989), which is based on allele frequencies at two different generations (see **Supplementary Method M2**). For this approach, only the SSR markers were used, as the SNP were more likely to be under selection.

#### Phenotypic Effects

To investigate the phenotypic effects of the selected alleles, we carried out an association analysis with two sets of data: (1) phenotypic assessments on single plants in mixed stands, i.e. within the diverse populations described so far, and (2) phenotypic assessments in pure stands of single genotypes. The latter reflects common crop stands of single genotypes and was assessed in standard plot field trials. Whereas in mixed stands every plant is surrounded by different genotypes, in pure stands the whole plot or field consists of one single genotype.

The effects of the marker loci in single plants in mixed stands were assessed on the tagged plants (see above) at SOF and WAF in generation 6 and 10, i.e. in four field trials in total. Data were analyzed with a mixed model for each marker locus separately:

(1) $$y_{ijk}=\mu +\;m_{j}+t_{k}+mt_{jk}+e_{ijk}$$

where *y_ijk_* is the response of the i-th plant, carrying the j-th allele, in the k-th trial, *μ* the grand mean, *m_j_* the effect of the j-th allele, *t_k_* the effect of the k-th trial, *mt_jk_* the allele–trial interaction, and *e_ijk_* are the residuals. The effects of the trial, allele–trial interaction, and the residuals were considered as random. As we aimed to calculate additive allele effects, only homozygous individuals for the respective marker were included.

The effects of the marker loci in pure stands, i.e. from the CCPs’ parent varieties, were assessed in common plot trials (3 years × 4 locations yielding overall 12 trials). First, LS-means were calculated with the mixed model

(2) $$y_{jln}=\mu +tr_{jn}+v_{l}+t_{j}+vt_{lj}+e_{jln}$$

where *y_jln_* is the response of the l-th variety in the n-th replicate block in the j-th trial, *μ* the grand mean, *tr_jn_* is the effect of the n-th replicate block nested in the j-th trial, *v_l_* is the effect of l-th variety, *t_j_* is the effect of the j-th trial, *vt_lj_* the variety–trial interaction, and *e_jln_* are the residuals. All effects except the variety effect were taken as random. Subsequently, the marker effect was assessed on the estimated LS-means with the following fixed model, where again one model was run for each marker locus:

(3) $$y_{jl}=\mu +m_{j}+e_{jl}$$

where *y_lj_* is the response of the l-th variety carrying the j-th allele, *m_j_* the effect of the j-th allele, and *e_jl_* are the residuals.

Significance of the marker effects (model 1 and 3) were tested by an F-test with Satterthwaite degrees of freedom in the mixed model. Additive allele effects of the frequent alleles were calculated as half of the estimated coefficient for the frequent allele.

To identify phenotypic traits responsible for selection and to investigate the different role in mixed and pure stand, we performed Pearson correlation between the changes in allele frequency from FND to generation 10 and the additive allele effects over all marker loci. Significance of the correlation was tested with a t-test.

The statistical analysis was performed in R, version 3.5.0 (R Core Team, 2018). Bootstrapping was performed with the *boot* package (Canty and Ripley, 2016), mixed models were fit with the *lme4* package (Bates et al., 2015), LS-means were calculated with the *emmeans* package (Lenth, 2018), and ANOVA of the marker effects was conducted with the *lmerTest* package (Kuznetsova et al., 2017).

## Results

### Gene Diversity

**Figure 2:**
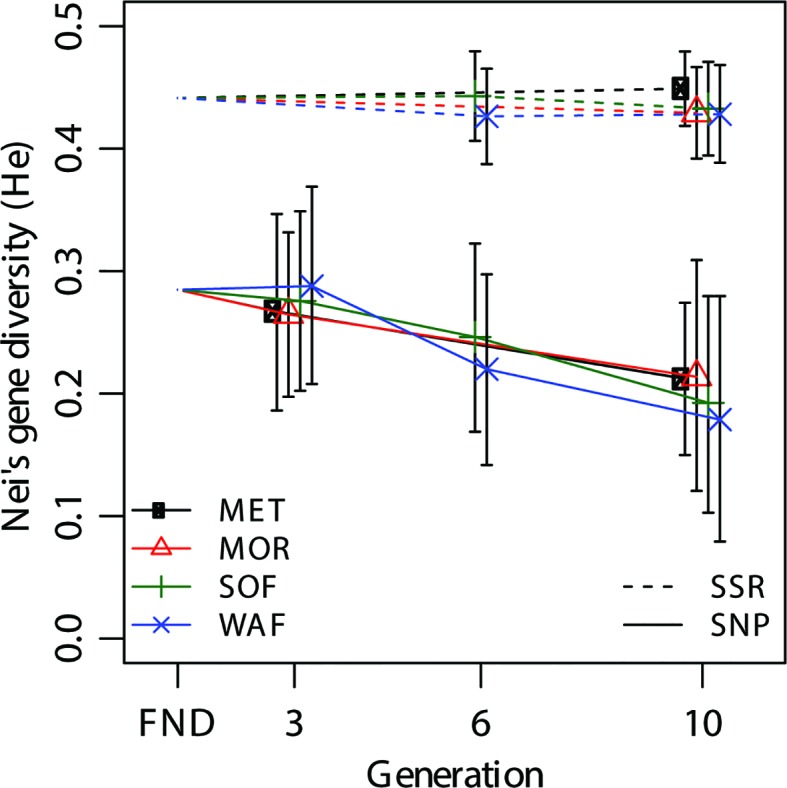
Change of Nei’s gene diversity (*H_e_*) over generations for the SNP and SSR marker sets at the four different locations Metfield (MET), Morley (MOR), Sheepdrove Organic Farm (SOF), and Wakelyns Agroforestry (WAF). FND indicates the founding population. Error bars are 95% CIs from bootstrapping over loci.

All alleles that were present in the FND were also found in all sampled populations, so none of the alleles were eliminated over 10 generations. The two marker types show different levels of gene diversity (**Figure 2**). However, as this measure depends on the allele frequencies, the marker types cannot be directly compared. While diversity in the SSR set remained constant at 0.44, it decreased from 0.28 to 0.20 in the SNP marker set (**Figure 2**). Estimates within generations did not differ between locations, indicating that the populations underwent similar changes at the four different locations.

### Genetic Differentiation

To investigate if the populations underwent a differential selection at the four locations, we estimated Weir and Cockerham’s *F_ST_*. *F_ST_* of zero indicates no differentiation and one represents fixation. The overall differentiation at generation 10 using all marker loci was *F_ST_* = 0.013 (95% CI: 0.008–0.018). Pairwise estimates (**Table 1**) ranged between *F_ST_* = 0.006 and *F_ST_* = 0.018. Both organically managed locations showed a lower estimate of *F_ST_* = 0.006 and a similarly small differentiation was found between SOF and MOR. To further test the differentiation due to management, we compared differentiation between both organically managed locations against both conventionally managed locations and compared this estimate to the pairwise groupings. The differentiation between management systems was higher [0.010 (0.005–0.015)] than both other groupings [WAF & MET vs. SOF & MOR: 0.005 (0.002–0.011) and SOF & MET vs. WAF & MOR: 0.005 (0.002–0.011)].

**Table 1:** Pairwise genetic differentiation at generation 10, measured by Weir and Cockerham’s *F_ST_* (above diagonal) with 95% CIs from bootstrapping over loci in parentheses (below diagonal).

	Conventional loations		Organic locations	
	MET	MOR	SOF	WAF
**MET**		0.011	0.015	0.018
**MOR**	(0.005–0.020)		0.006	0.012
**SOF**	(0.008–0.023)	(0.003–0.011)		0.006
**WAF**	(0.007–0.031)	(0.006–0.020)	(0.001–0.012)	

Metfield (MET) and Morley (MOR) were conventionally managed; Sheepdrove Organic Farm (SOF) and Wakelyns Agroforestry (WAF) were organically managed.

### Effective Population Size

Effective population size (*N*_e_) was estimated using the overall genetic differentiation in generation 10 and marker loci not under differential selection. These excluded three loci (*Ppd-B1*, *gwm165-4B*, and *gwm46-7B*), which were identified to be under differential selection (P < 0.05 and *F_ST_* higher than average, also see **Figure S1**). Using the remaining loci, the overall genetic differentiation was estimated as *F_ST_* = 0.009 (0.006–0.013), resulting in *N_e_* = 221 (153–332). The temporal method produced an estimate of *N_e_* = 140, averaged across all comparisons for the SSR maker loci (**Table S2**). Here, we report only the estimate from the SSR markers, as the investigation on gene diversity already indicates that selection took place in the SNP marker set, and absence of selection is a prerequisite for the temporal method.

### Changes in Allele Frequency

**Figure 3:**
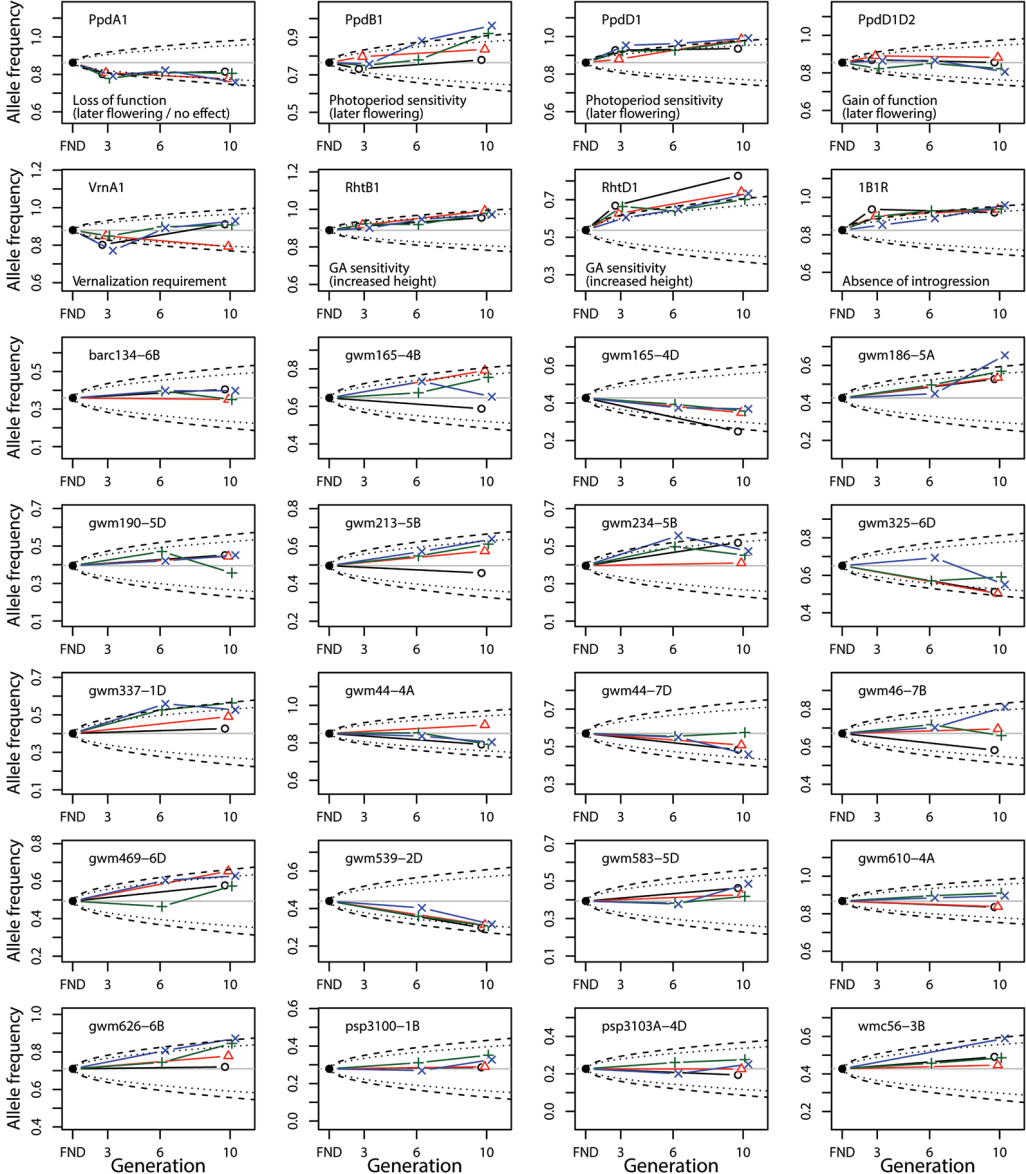
Change of allele frequency in the composite cross populations (CCP) starting from the estimated allele frequency of the virtual founding population (FND). The allele frequency is shown for the frequent allele in the FND population. The different colors denote the allele frequencies in the populations at the different locations (black: MET, red: MOR, green: SOF, blue: WAF). The dashed and dotted lines indicate the 95% CI of the allele frequency expected under pure genetic drift given an *N_e_* = 150 and *N_e_* = 250, respectively. For the SNP marker loci (top two rows), the function of the frequent allele is given.

Given two estimates for *N_e_*, we inspected visually if the observed changes in allele frequency were greater than expected under pure genetic drift for *N_e_* = 150 and *N_e_* = 250 (**Figure 3**). Instead of using only one final estimate, we used these two boundaries which allows comparisons of the size of expected genetic drift under different *N_e_*.

Overall, the observed changes of allele frequency have could occured due to genetic drift alone, even for the smaller *N_e_* = 150. However, six of the gene-based markers (*Ppd-A1*, *Ppd-B1*, *Ppd-D1*, *Rht-B1*, *Rht-D1*, and *1B.1R*) showed consistent changes over generations, which were greater than expected under genetic drift. Except for *Ppd-B1*, the direction of selection was the same across locations and towards the wild-type (WT) allele.

For *Ppd-B1* selection was only found at SOF and WAF. Furthermore, many SSR marker loci also showed similar selection for the same allele at all four locations (most notably *gwm165-4D*, *gwm186-5A*, *gwm539-2D*). The two loci which were identified to be under differential selection, *gwm165-4B* and *gwm46-7B*, showed the greatest variation at generation 10, with selection at MET being different to the other locations.

To test if selection was generally towards the similar direction at all four locations, we correlated the changes in allele frequency between FND and generation 10 at the different locations. **Table 2** shows that the changes were highly correlated (P < 0.001) between the different locations. The strongest correlation (r = 0.82) was between SOF and WAF, the two organically managed locations.

**Table 2:** Pearson correlation coefficients (above diagonal) of the difference of allele frequency between generation 10 at the different locations and the founding population (FND), with significance level indicated below the diagonal (***P < 0.001, based on a t-test with df = 26) for the four different locations Metfield (MET), Morley (MOR), Sheepdrove Organic Farm (SOF), and Wakelyns Agroforestry (WAF).

	MET	MOR	SOF	WAF
**MET**		0.70	0.64	0.70
**MOR**	***		0.77	0.74
**SOF**	***	***		0.82
**WAF**	***	***	***	

### Phenotypic Effects of the Selected Alleles

**Figure 4:**
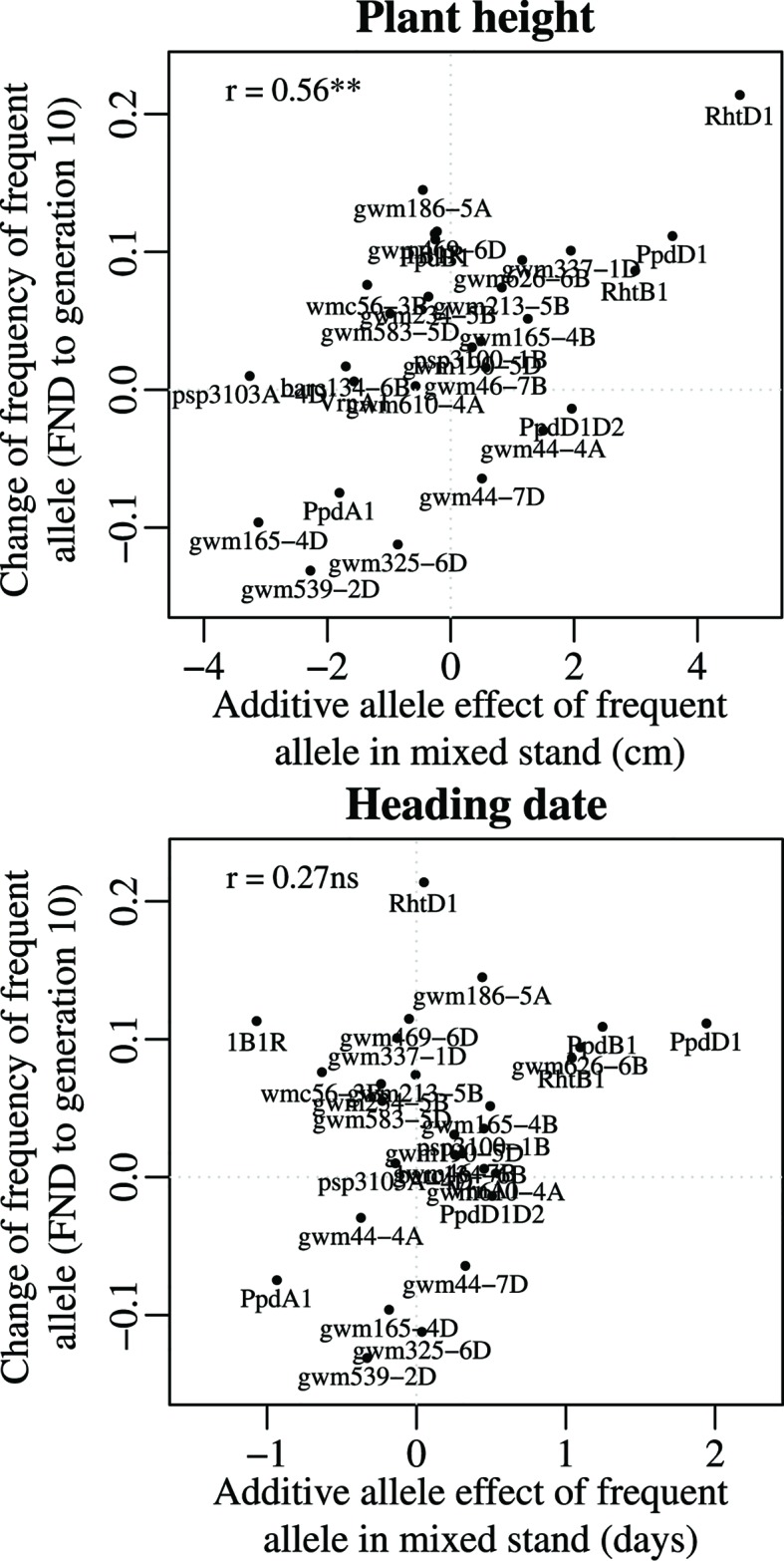
Relationship between the additive allele effect on plant height and on heading date and the temporal change in allele frequency from the virtual founding population (FND) to the allele frequency at generation 10 (averaged over all four locations). For plant height, the significant correlation indicates that those genes with a stronger effect on plant height (such as *Rht-D1*) tended to have a more pronounced selection over time, demonstrated by the high change in allele frequency.

To investigate the phenotypic effects of the selected alleles, we correlated the changes of allele frequency with the additive allele effect in the mixed crop stands. Only plant height showed a significant correlation (P < 0.01) to the overall changes of allele frequency between FND and generation 10 (**Table 3** and **Figure 4**). This relation indicates that height increasing alleles were under positive selection. This was true at both *Rht* homoeoloci. Interestingly, also *Ppd-A1* and *Ppd-D1* showed a significant effect on plant height (see F-Test for marker trait associations in **Tables S3** and **S4**), again with the height increasing allele under positive selection (**Figure 4**). Furthermore, at three SSR marker loci (*gwm165-4D*, *gwm325-6D*, and *gwm539-2D*), where there was consistent selection for the rare allele at all locations, the rare allele had a significant and increasing effect on plant height.

**Table 3:** Pearson correlations coefficient between the additive allele effects for the named traits measured in single plants in mixed stands and the change in allele frequency from FND to the average allele frequency at generation 10 (overall), and to the allele frequency at each location; *, **, *** denote significant correlation at P < 0.05, P < 0.01, and P < 0.001, respectively; ns indicates non-significance (P > 0.05).

Additive allele effect on	Overall	MET	MOR	SOF	WAF
**Plant height**	0.56**	0.49**	0.68***	0.50**	0.36 ns
**Tillers per plant**	0.31 ns	0.03 ns	0.23 ns	0.46*	0.39*
**Grain number per tiller**	−0.10 ns	−0.08 ns	−0.04 ns	−0.07 ns	−0.15 ns
**Thousand grain weight**	0.23 ns	−0.02 ns	0.25 ns	0.42*	0.19 ns
**Grain weight per tiller**	0.18 ns	<0.01 ns	0.24 ns	0.34 ns	0.11 ns
**Harvest index**	−0.36 ns	−0.22 ns	−0.36 ns	−0.40*	−0.31 ns
**Heading date**	0.27 ns	0.09 ns	0.26 ns	0.36 ns	0.27 ns

The relation between the additive allele effect on plant height and the change of allele frequency was also significant (P < 0.01) at each location except at WAF (**Table 3**). At both organic locations, SOF and WAF, the change in allele frequency was significantly correlated with an increasing additive effect of the selected alleles on the number of tillers per plant (**Table 3** and **Figure S2**). However, it is difficult to identify single markers which are responsible for this significant correlation (**Figure S2**).

Relationships between the additive allele effects and yield components (grain number per tiller, thousand grain weight, and grain weight per tiller) were not significant, neither were harvest index and heading date (**Table 3**).

Alleles often have pleiotropic effects, which can also result in the correlation between traits. As an example, we calculated the correlations between plant height and various agronomically important traits. We compared these correlations among different traits for single plants in mixed stands compared to pure stands (**Table 4**). Increased plant height was associated with decreased grain number per tiller in pure stands, while this effect was not significant in the mixed stand. Similarly, plant height was negatively associated with grain weight per tiller in pure stands, while in mixed stands the relation was reversed, though not significant. For all three yield components (tillers per plant, grain number per tiller, and thousand grain weight), the correlation with plant height was smaller (i.e. more negative or less positive) for the pure stands than for the mixed stands. In the mixed stands, where different genotypes directly competed, plant height was needed more to generate yield than in the pure stands.

**Table 4:** Correlations between the additive allele effects on plant height and allele additive effects on various yield components and heading date, for single plants within mixed stand and for pure stands of single genotypes; (NA): data not available; * and *** denote significant correlations at P < 0.05 and P < 0.001, respectively; ns indicates non-significance (P > 0.05).

	Additive allele effects on plant height	
	In single plants within mixed stands	In pure stands of single genotypes
**Tillers per plant**	0.19 ns	−0.24 ns
**Grain number per tiller**	−0.15 ns	−0.78***
**Thousand grain weight**	0.37 ns	0.23 ns
**Grain weight per tiller**	0.29 ns	−0.78***
**Harvest index**	−0.77***	−0.93***
**Heading date**	0.47*	(NA)
**Grain yield**	(NA)	−0.70***

Further analysis showed that grain yield (the product of the three yield components) in pure stand was mostly dependent on grain number per tiller (data not shown). However, natural selection acted towards a decreased grain number per tiller (**Table 3**).

## Discussion

### Gene Diversity

While gene diversity did not decrease at the SSR markers in all four independent populations, gene diversity decreased at the SNP markers, with equal magnitude in all populations (**Figure 2**). The fact that gene diversity remained constant at the SSR markers indicates that little or no selection had taken place on loci tracked by these markers. In contrast, the decrease in gene diversity at the SNP markers suggests that selection on these functional markers did take place, and that selection, overall, was of similar magnitude and direction at all locations. As absolute values of *H_e_* were dependent on the genetic composition, they cannot be directly compared to other studies where populations of different composition were used. However, Raggi et al. (2016) using 22 SSR markers also found no decrease in *H_e_* in a CCP of barley that had evolved for 13 years. More generally, our results confirm that overall genetic diversity in evolving wheat populations appears to be maintained to a large degree unless there is a strong specific selection force [e.g. (Paillard et al., 2000)].

### Effective Population Size

Estimation of effective population size (*N_e_*) is crucial for the identification of loci with changes in allele frequency greater than expected under pure drift. The method based on the genetic differentiation (*F_ST_*) produced a higher estimate than the temporal method, using only the SSR markers (220 *vs.* 140, **Table S2**). The estimates from the temporal method based on the SNP markers showed an even smaller estimate (50, averaged over all comparisons), which is most likely due to selection taking place on these loci. As selection also took place on some of the SSR loci, the estimate from the temporal method also appears biased towards low estimates.

The values estimated in our study are higher than those reported by Rhoné et al. (2010) for different wheat populations grown over several generations in France, where estimates for *N*_e_ were 33, 114, and 118 at three locations. Other estimates from wheat populations in France, with *N*_e_ = 311 (Thépot et al., 2015) and *N*_e_ = 42 to *N*_e_ = 208 (Enjalbert et al., 1999) were closer to the values estimated here.

### Differentiation Between Locations

One of our main aims was to evaluate whether genetic differentiation occurred over 10 generations for wheat CCPs grown in contrasting environments with very different management. To assess the strength of population differentiation we took values between 0 and 0.05 as a general convention for little differentiation (Wright, 1978; Hartl and Clark, 1997). According to this convention, the values observed in this study are very low, sometimes not even significantly different from zero. Even when investigating *F_ST_* values for the single loci, *F_ST_* values were still below 0.05 (data not shown).

Although the locations in our study differed in management (organic *vs.* conventional), resulting in strong variation in average yield level (around 9.5 t/ha at the conventional locations, and 5.3 t/ha at the organic locations (Jones et al., 2010; Döring et al., 2015)), no consistent genetic differentiation regarding the management practices could be detected. In fact, pairwise comparisons between locations showed low values for genetic differentiation (**Table 1**), and selection was similar between locations, as indicated by the highly significant correlations between changes of allele frequencies (**Table 2**). In addition, markers diagnostic for genes of known function such as plant height and photoperiod sensitivity were not affected differently at the four studied locations (**Figure 3**).

Four possible reasons for this lack of consistent genetic differentiation between the four locations are: 1) the number of generations was not sufficient to allow selection to exert a measurable effect on the genetic composition of the CCPs; 2) some loci did exhibit environment specific selection but markers to detect these changes were not included in this study; 3) even though management is different between the locations, effective environmental conditions might be quite similar; and 4) yearly weather fluctuations superimposed the environmental conditions at the specific locations. Regarding the number of generations required to observe adaption to local conditions, Allard (1988) found that first changes in their populations took place within the first 5 to 10 generations. Thus, such changes should have been observable in our experiment. A higher marker density could have possibly identified loci that were under differential selection, which could be used in future to detect loci for local adaption. However, yearly weather fluctuations leading to yearly variation in disease pressure and nutrient availability could probably superimpose local differences in growing conditions and hinder local adaption.

### Differentiation Over Time Across All Locations

The more exciting outcome of our analysis was the detection of a clear selection signature in terms of temporal differentiation. At all four locations, genetic changes were observed in the same direction, in particular for the alleles linked to increased plant height and later flowering time (**Table 3**). These changes over time without any population differentiation can be summarized for most cases as selection towards wild-type. At the five loci for which significant changes of allele frequencies could be detected, there was selection for the wild-type alleles and against the mutant alleles that were introduced during the twentieth century through the implementation of systematic wheat breeding.

The two loci that have undergone the greatest change of allele frequencies were genes controlling height (*Rht-B1* and *Rht-D1*) (**Figures 3** and **4**), suggesting that plant height has been a driving force in the evolutionary process of the investigated CCPs. This observation is supported by a study by Raquin et al. (2008), who found an increase of *Rht-B1* allele frequency in an experimental population of winter wheat from 0.66 in the initial generation to near-complete extinction of the dwarfing allele after 17 generations. The semi-dwarfing alleles at both loci were of major importance during the Green Revolution (Borlaug, 1983). Currently, 58% of all European winter wheat varieties contain the *Rht-D1b* allele and 7% contain the *Rht-B1b* allele (Zanke et al., 2014). The selection for increased height can independently be observed in several other markers as well (**Figure 4** and **Table 3**). These genetic effects confirm and explain the phenotypic observations on the same populations, which, in an earlier study, led to the conclusion that already in the third year of development, the wheat CCPs were almost 10 cm taller than the mean of the parents (Döring et al., 2015).

The selection for increased height may mainly be explained by competition for light. At the population level, competition in a genetically diverse plant stand selects for taller intra-specific competitors. It is therefore expected that genotypes with increased height are selected over time, and this is confirmed by our study. However, competition for light may not be the only driver for selection against the so-called dwarfing genes. In particular, the dwarfing genes *Rht-B1b* and *Rht-D1b* confer effects of reduced early vigor through shorter coleoptiles and reduced vigor in young plants. More generally, the selection observed in this study can be characterized as going towards more vegetative growth and more competitive ability, which could reduce yield potential (Denison, 2012).

The observed selection for increased height suggests that adaptation took place towards growing in a mixed stand population rather than to environmental conditions. This is because the dwarf genotypes, when grown together with taller neighbors will produce a reduced number of progenies, and thus be selected against over time. Thus, while the performance of a single plant in a mixed stand is determined by its competition with its neighbors (e.g. through plant height), this is not the case in the pure stand.

The selection for the wild-type alleles at the genes *Ppd-B1* and *Ppd-D1* restores photoperiod sensitivity, as the mutant allele at both genes cause insensitivity to photoperiod (day length neutrality) and early heading. Again, these genes and alleles were very important for the Green Revolution (Borlaug, 1983), allowing wide adaptation. Thus, also for the two *Ppd-1* genes, selection seems to have happened towards wild-type and against alleles that are important in modern agricultural production. It should be noted also that the majority of UK wheat varieties are photoperiod sensitive so these results are also a reversion to UK type. Interestingly, the mutant allele of *Ppd-1* is also responsible for a further shortening of plant height, due to the temporal shortening of vegetative growth (Börner et al., 1993). Accordingly, we observed a significant correlation between allele effects on plant height and heading date (**Table 4** and **Figure 4**). Because of the pleiotropic effects it remains still open what the driving factor for the changes in allele frequency of the *Ppd* genes were in the wheat CCPs. In particular, it remains open whether later flowering itself conveyed a fitness advantage to individuals within the evolving wheat populations.

The selection for the wild-type form can also be hypothesized for the *1B/1R* marker. This marker identifies the translocation from rye into wheat, which is widespread in many breeding programs, and mostly originates from the rye variety Petkus (Schneider and Molnár-Láng, 2009). In the studied populations, selection occurred against the translocation, even though it is assumed that the introgression confers increased disease resistance (Heslop-Harrison et al., 1990) and should thus also lead to improved fitness under disease pressure.

As our observation is that the major pattern of selection is the result of selection for wild-type alleles, it may be suggested that evolutionary plant breeding approaches can be improved by fixing these alleles, so that negative selection cannot occur. Consequently, individual plants within the diverse population would not invest resources on competitive behavior and selection would then be directed towards prevailing local growing conditions. However, traits that are linked to the competitive ability of the plant, such as plant height, are governed by a large number of genes (Zanke et al., 2014). It is therefore unlikely that competition within a population can be fixed genetically without substantially reducing genetic diversity. Since it seems impossible to create populations that are completely free of any trade-offs, future research will be needed to address the question which trade-offs show the greatest opportunities for developing multifunctional, and potentially adaptive, CCPs.

## Data Availability Statement

All datasets generated for this study are included in the **Supplementary Material**.

## Author Contributions

SK performed data analysis and led writing of manuscript. TD organized field work and contributed to manuscript. HJ contributed to early experimental plans. MW and JS conceived project and won funding. LW supported data analysis. ML-W carried out molecular analysis. SG organized project, molecular work, and contributed to writing of manuscript.

## Funding

The work was funded under the LINK scheme with financial support from the UKs Department of Environment Food and Rural Affairs (Defra), the Biotechnology and Biological Sciences Research Council (BBSRC), and the Agriculture and Horticulture Development Board (AHDB). The project title was: Adaptive winter wheat populations: development, genetic characterization and application (LK0999).

## Conflict of Interest

The authors declare that the research was conducted in the absence of any commercial or financial relationships that could be construed as a potential conflict of interest.

## Supplementary Material

Presentation 1 Supplementary Figures and Tables

Presentation 2 Supporting Method M1

Datasheet 1 All data generated for this study, as used in the analysis
