# Supplementary material for: Natural Selection Towards Wild-Type in Composite Cross Populations of Winter Wheat
Source: Front Plant Sci. 2020 Feb 25;10:1757. doi: 10.3389/fpls.2019.01757 (PMC7053226; doi:10.3389/fpls.2019.01757)
Supplement: Presentation 2 — Supporting Method M1 [file Presentation_1.pdf]

Supporting Information

Supporting Methods

M2: Estimation of Ne with the 'temporal method'

As a second method to estimate N_e, we used the 'temporal method' as first developed by Waples (1989), which is based on the allele frequencies in two generations. We first calculated the standardized variance of gene frequency change, $\widehat{F_c}$, for each locus, assuming sampling before reproduction (eq. 8 in Waples (1989)):

$$\widehat{F_c} = \frac{1}{K}\sum_{i=1}^{K}\frac{(x_i-y_i)^2}{(x_i-y_i)/2-x_i y_i},$$

where K is the number of alleles, x_i and y_i are the frequencies of the i-th allele in the two different generations. As all loci had two alleles (see Material and Methods in the main section) the genomewide-estimate ($\overline{F_c}$) was calculated as the mean over all per loci estimates. The number of sampled individuals was calculated as harmonic mean over loci (Nei and Tajima, 1981) and Ne was calculated as

$$N_e = \frac{t}{2[\overline{F_c}-\frac{1}{2S_1}-\frac{1}{2S2}]},$$

where t is the number of generations, and S_1 and S_2 are the numbers of sampled individuals in the first and in the second generation, respectively. 95% confidence intervals (CI) for the estimates of N_e were derived, assuming that $\frac{n\overline{F_c}}{E(\overline{F_c})}$ is χ^2 –distributed with n degrees of freedom and n being the numbers of loci (Lewontin and Krakauer, 1973)

$$95\%\ CI\ (\overline{F_c}) = \{\frac{n\overline{F_c}}{\chi^2(0.025,n)};\frac{n\overline{F_c}}{\chi^2(0.975,n)}\}$$

and subsequent calculation the 95% CIs of N_e.

Supporting Tables

Table S 1: Chromosome, number of alleles scored in the parental genotypes and parental genotypes carrying the most frequent alleles. A one indicates that this parental genotype carries the most frequent allele for the given locus.

**Locus**	**Marker set**	**Chromosome**	**Number of alleles**	**Bezostaya**	**Buchan**	**Cadenza**	**Claire**	**Deben**	**Hereward**	**HTL**	**Mercia**	**Monopol**	**MWidgeon**	**Norman**	**Option**	**Pastiche**	**Renan**	**Renesansa**	**Soissons**	**Spark**	**Tanker**	**Thatcher**	**Wembley**
B1R_1B	SNP	1B	2	1	0	1	1	1	1	0	1	1	1	1	1	1	1	1	1	1	0	1	1
Ppd-A1Cdex_2A	SNP	2A	2	1	1	1	1	1	1	1	1	1	0	1	1	1	1	1	1	0	1	1	1
Ppd-B1L5_2B	SNP	2B	2	0	1	1	1	1	1	1	1	1	1	1	1	1	0	1	0	1	1	0	0
Ppd-D1_2D	SNP	2D	2	0	1	1	1	1	1	1	1	1	1	1	1	1	1	0	0	1	1	1	1
Ppd-D1D2_2D	SNP	2D	2	1	1	1	1	0	1	1	1	1	1	0	1	0	1	1	1	1	1	1	1
Rht-B1_4B	SNP	4B	2	1	1	1	1	1	1	1	1	1	1	1	1	1	0	1	0	1	1	1	1
Rht-D1_4D	SNP	4D	2	1	0	1	0	0	0	1	1	1	1	0	0	0	1	1	1	1	0	1	0
VrnA1prom_5A	SNP	5A	2	1	1	0	1	1	1	1	1	1	1	1	1	1	1	1	1	1	1	0	0
barc134_6B	SSR	6B	7	0	1	0	0	1	1	0	0	0	0	1	1	0	0	0	0	0	1	0	1
gwm165_4B	SSR	4B	4	0	1	1	0	1	1	1	1	0	1	1	0	1	0	0	0	1	1	1	1
gwm165_4D	SSR	4D	4	1	1	0	0	1	0	0	1	0	0	1	1	1	0	1	0	0	0	0	1
gwm186_5A	SSR	5A	5	0	1	0	1	1	1	0	0	1	1	0	1	0	0	0	1	0	0	0	0
gwm190_5D	SSR	5D	4	1	0	1	0	0	1	0	0	0	1	0	0	0	1	1	1	1	0	0	0
gwm213_5B	SSR	5B	6	0	1	0	1	1	1	1	1	0	1	1	0	1	0	0	0	0	0	1	0
gwm234_5B	SSR	5B	5	0	1	0	1	1	0	1	1	0	0	0	1	1	0	0	0	0	0	0	0
gwm325_6D	SSR	6D	4	1	1	0	1	1	1	1	1	1	0	1	0	1	1	0	0	1	0	0	1
gwm337_1D	SSR	1D	5	0	0	1	0	1	0	1	1	0	1	1	0	1	1	0	0	0	0	0	0
gwm44_4A	SSR	4A	2	1	1	1	0	0	1	1	1	1	1	1	1	1	1	1	1	1	1	1	1
gwm44_7D	SSR	7D	4	0	1	1	0	0	1	0	0	1	1	1	0	1	1	0	1	1	1	0	1
gwm46_7B	SSR	7B	4	0	1	1	1	1	0	1	1	0	1	1	0	0	0	1	0	1	1	1	0
gwm469_6D	SSR	6D	5	0	1	1	0	1	1	1	0	1	0	0	1	0	0	0	0	1	0	1	1
gwm539_2D	SSR	2D	5	0	0	0	1	1	0	0	1	0	0	1	0	0	0	0	1	0	1	1	1
gwm583_5D	SSR	5D	4	1	0	0	0	0	1	1	1	1	0	0	1	0	1	0	0	1	0	0	0
gwm610_4A	SSR	4A	2	1	1	1	1	1	1	1	1	1	1	1	1	1	1	1	0	1	1	0	0
gwm626_6B	SSR	6B	2	0	1	1	1	1	1	1	1	1	1	1	1	0	0	0	0	1	1	0	0
psp3100_1B	SSR	1B	7	0	0	0	1	1	0	0	0	0	1	1	0	1	0	0	0	0	0	0	0
psp3103A_4D	SSR	4D	7	0	0	0	0	1	1	0	0	0	0	1	1	1	0	0	0	0	0	0	0
wmc56_3B	SSR	3B	5	0	1	0	1	1	0	1	0	0	0	1	1	0	1	0	0	0	0	0	0

Table S 2: Estimates of effective population size based on the temporal method as described in supporting methods (M1).

Marker set	Paired generations	FND vs generation 6		FND vs generation 10				Generation 6 vs 10	
	**Site**	**SOF**	**WAF**	**MET**	**MOR**	**SOF**	**WAF**	**SOF**	**WAF**
SSR	Ne	210	128	156	165	146	88	144	74
	95% CI	[94-393]	[58-237]	[73-280]	[77-293]	[68-259]	[41-154]	[62-288]	[33-139]
SNP	Ne	78	47	56	40	43	29	57	56
	95% CI	[25-226]	[15-135]	[17-141]	[12-99]	[13-109]	[9-73]	[15-137]	[14-137]

Table S 3. F-Test of the marker main effect in single plants in mixed stand.

Locus	Plant height	Tillers per plant	Grain number per tiller	Thousand grain weight	Grain weight per tiller	Harvest index	Heading date
**PpdA1**	19.5***	0	1.4	0.9	0.7	5.4*	24.7***
**PpdB1**	0	0.2	0.1	1.5	0.6	0.1	4.6
**PpdD1**	4	3.3	0	3.1	0.3	0.7	3.9
**PpdD1D2**	13.0*	0.5	0	5.1	2.1	0.4	3.4
**VrnA1**	6.7	0.1	0.1	0.1	0.1	0.1	0.7
**RhtB1**	8.4	0.4	1.3	5.7	2.6	0.1	1.5
**RhtD1**	29.3*	1.6	4.5*	0	2.4	9.9	0
**1B1R**	0.1	0.1	2	0	0.7	0.8	2.3
**barc134-6B**	5.6	0.3	0	2.3	0.4	3.7	3.5
**gwm165-4B**	1.9	1.4	0.1	30.6***	3	0.8	4.1*
**gwm165-4D**	87.0***	0.3	2.3	2.5	0.1	5.1	0.4
**gwm186-5A**	1.2	0.1	1.6	0.1	1.6	1.1	1.2
**gwm190-5D**	1.1	0.1	0.6	0.1	0.5	0.3	1.3
**gwm213-5B**	5.4*	5.7	2.2	12.8*	0.2	10.9*	0
**gwm234-5B**	0.3	0.1	0.5	0.1	0.2	3.2	2
**gwm325-6D**	3.4	2.7	2.1	0.4	2.5	1.9	0.1
**gwm337-1D**	29.3*	1.3	0	19.1*	1	3.4	0.1
**gwm44-4A**	2.5	2	4.9*	0	2.6	0	1.3
**gwm44-7D**	2.2	1.4	0.3	5.2	2.6	0.1	4.2*
**gwm46-7B**	2.3	2.4	1.1	10.1	0	0.7	1.9
**gwm469-6D**	0.2	0	0.3	6.8	2.9	2.3	0.1
**gwm539-2D**	40.8***	2.5	0.7	0	1.4	1.6	2.9
**gwm583-5D**	8.8**	1.1	0.6	16.2*	0.3	0	1.5
**gwm610-4A**	0.2	0.9	0.2	0.2	0.4	0.1	1.3
**gwm626-6B**	2.6	0.4	0.2	2.9	0	0.2	23.0***
**psp3100-1B**	1.8	0.7	0	11.0*	3	0.3	2.2
**psp3103A-4D**	48.0**	0.9	0.7	0.2	0.9	3.4	0.6
**wmc56-3B**	9.4	0.2	1.7	4.2	0.2	0.2	16.3***

*,**,*** denotes $P<0.05$,$P<0.01$,and $P<0.001$, respectively, of the F-Test of marker main effect in the ANOVA

Table S 4. F-Test of the marker main effect in single genotype pure stand.

Locus	Plant height	Tillers per plant	Grain number per tiller	Thousand grain weight	Grain weight per tiller	Harvest index	Grain yield
**PpdA1**	4.8*	0.2	1.3	0.1	0.8	3.4	0.1
**PpdB1**	1.2	1	2.1	0.9	1.1	0.3	1.5
**PpdD1**	0.1	12.0**	0	0.8	0.6	0.5	1.2
**PpdD1D2**	0.3	0.4	0.5	0.2	1.5	0.2	1.5
**VrnA1**	2.1	0.4	0.8	1.3	3.9	1	1.7
**RhtB1**	0.2	0.2	1.6	1	0.6	0.1	0.2
**RhtD1**	9.0**	3.6	15.5**	2.2	6.2*	9.0**	8.2*
**1B1R**	1.3	2.9	0	0	0.1	0.2	0.1
**barc134-6B**	5.7*	6.3*	8.9**	2	3.8	6.7*	5.9*
**gwm165-4B**	0.2	6.6*	0.1	0.9	0.7	0.6	0.1
**gwm165-4D**	2.6	0	7.8*	0	8.7**	5.0*	1.2
**gwm186-5A**	0.2	0.1	1.3	0.6	0.3	0.1	1.8
**gwm190-5D**	0.4	3.8	2.3	1.2	0.5	0.5	1.9
**gwm213-5B**	0.4	0.4	0.1	0	0.1	0.9	0
**gwm234-5B**	2.2	3.1	2.9	1.3	0.7	1.5	5.7*
**gwm325-6D**	0.5	0.5	0	0	0	0	1.1
**gwm337-1D**	0.7	0.3	1.2	1.8	0.1	1.5	0
**gwm44-4A**	0.4	1.2	2.4	0.7	0.9	1.3	4.8*
**gwm44-7D**	0.2	0.2	0.3	0	0.1	0.2	0
**gwm46-7B**	0.3	2.2	0	0.5	0.3	0.1	0.1
**gwm469-6D**	0.4	7.8*	0.4	3.4	4.8*	1	0
**gwm539-2D**	0.3	1.4	2.5	2.8	0.2	2.6	4
**gwm583-5D**	0	0.1	0.7	0.1	0.6	0.4	0
**gwm610-4A**	1.2	0	0.6	0.6	2.6	0.1	0.2
**gwm626-6B**	0.3	5.4*	1.2	3.5	0	0	2.8
**psp3100-1B**	0.3	0	0.5	0.4	1.6	0.1	0.5
**psp3103A-4D**	1.5	0.4	2	0.2	1.4	1.1	2.3
**wmc56_3B**	0.9	4.6*	0.1	0	0	0.6	2.8

*,**,*** denotes $P<0.05$,$P<0.01$,and $P<0.001$, respectively, of the F-Test of marker main effect in the ANOVA

Supporting Figures

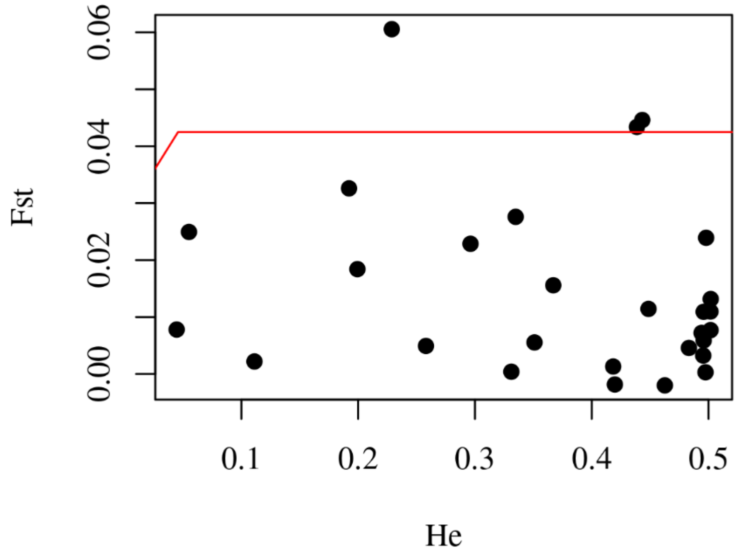

Fig. S1 F_{st} values at generation 10 comparing all 4 bread wheat CCP populations plotted against expected heterozygosity (H_e). The red line indicates the 95% null distribution quantile from the island model simulation with *Arlequin* based on 20,000 simulations with 100 demes (see Material and methods). The three dots above the red line represent the three loci identified as under selection (P<0.05).

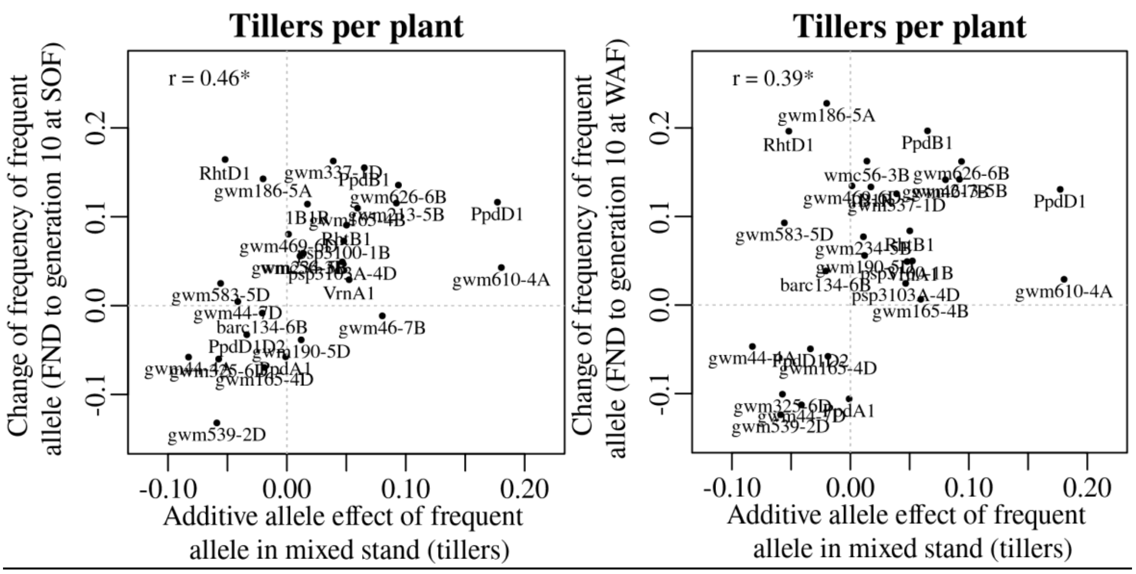

Fig. S2. Relation of the additive allele effect on tillers per plant in single plants in mixed stands to the change in allele frequency at SOF (left) and WAF (right).
