# Supplementary material for: Natural Selection Towards Wild-Type in Composite Cross Populations of Winter Wheat
Source: Front Plant Sci. 2020 Feb 25;10:1757. doi: 10.3389/fpls.2019.01757 (PMC7053226; doi:10.3389/fpls.2019.01757)
Supplement: Datasheet 1 — All data generated for this study, as used in the analysis [file Presentation_2.pdf]

Prediction of Ne from Fst

Simulation

Creation of the initial population

An initial population effective population size N_e has initial allele frequency p_0. Only one locus with two alleles A and B is considered, with allele frequency p_0 of allele A and $p_0 - 1$ of allele B. The allele frequency of A will be denoted a p. Genotypes are created in hardy Weinberg equilibrium:

$$p_{AA} = p_0^2$$
$$p_{AB} = 2p_0(1 - p_0)$$
$$p_{AA} = (1 - p_0)^2$$

```
Ne = 200
p0 = 0.5
pop_start <- c(rep("AA",p0^2*Ne),
               rep("AB",2*p0*(1-p0)*Ne),
               rep("BB",(1-p0)^2*Ne))
# genotype frequencies
table(pop_start) /Ne
```

```
## pop_start
##   AA   AB   BB
## 0.25 0.50 0.25
```

```
# allele frequencies
table(strsplit(paste(pop_start,collapse=""),""))/(2*Ne)
```

```
##
##   A   B
## 0.5 0.5
```

Sampling the sub-populations

From the first generation r independent sup-populations are created that evolve for t generations. Each sub-populations also has effective population size N_e. It is assumed that there is no migration between the sub-populations.

```
r = 500
t = 50
```

Each sup-population evolves as a Fisher-Wright population, i.e. in each the first allele is randomly sampled from the parents and the second allele from an infinite pollen cloud of alleles (with replacement). In the sampling function the allele frequency of A (p) and the genotype frequencies (p_{AA}, p_{AB}, p_{BB}) in each generation is recorded.

```
samplingfunc <- function(pop_start,Ne,t)
{
  pop <- pop_start
  pvec <- c()
  AAvec <- c()
```

```
  ABvec <- c()
  BBvec <- c()
  for (ti in 1:t)
  {
    # sample one random allele from each individual
    firstallelepos <- sample(1:2,Ne,replace=T)
    firstallele <- substr(pop,firstallelepos,firstallelepos)
    # sample second allele from the infinite pollen cloud, i.e. from all alleles with replacement
    secondallele <- sample(strsplit(paste(pop,collapse=""),"")[[1]],Ne,replace=T)
    pop <- paste(firstallele,secondallele,sep="")
    # extract allele frequency
    pvec <- c(pvec,sum(strsplit(paste(pop,collapse=""),"")[[1]]=="A")/(2*Ne))
    # and genotype frequencies
    AAvec <- c(AAvec,sum(pop=="AA")/Ne)
    ABvec <- c(ABvec,sum(pop=="AB"|pop=="BA")/Ne)
    BBvec <- c(BBvec,sum(pop=="BB")/Ne)
  }
  return(list(pvec,AAvec,ABvec,BBvec))
}
```

The results for each replicated sub-populations is recorded in a list and subsequently turned into a dataframe, where each column contains the allele frequencies of one population over the generations.

```
# use parallalization
library(parallel)
# with ncores
ncores=6
# run actual sampling process
poplist <- mclapply(1:r,function(x) samplingfunc(pop_start,Ne,t),mc.cores=ncores)
ptab  <- t(data.frame(matrix(unlist(lapply(poplist,function(x)x[[1]])), nrow=r, byrow=T)))
AAtab  <- t(data.frame(matrix(unlist(lapply(poplist,function(x)x[[2]])), nrow=r, byrow=T)))
ABtab  <- t(data.frame(matrix(unlist(lapply(poplist,function(x)x[[3]])), nrow=r, byrow=T)))
BBtab  <- t(data.frame(matrix(unlist(lapply(poplist,function(x)x[[4]])), nrow=r, byrow=T)))
```

Inspect the change in allele at sub-population 3. Horizontal lines indicate the starting allele and genotype frequencies, respectively.

```
par(mfrow=c(1,4))
plot(ptab[,3],xlab="Generation",ylab="Allele frequency of A (p)",ylim=c(0,01));abline(h=0.5)
plot(AAtab[,3],xlab="Generation",ylab="Genotype frequency of AA",ylim=c(0,01));abline(h=0.25)
plot(ABtab[,3],xlab="Generation",ylab="Genotype frequency of AB&BA",ylim=c(0,01));abline(h=0.5)
plot(BBtab[,3],xlab="Generation",ylab="Genotype frequency of BB",ylim=c(0,01));abline(h=0.25)
```

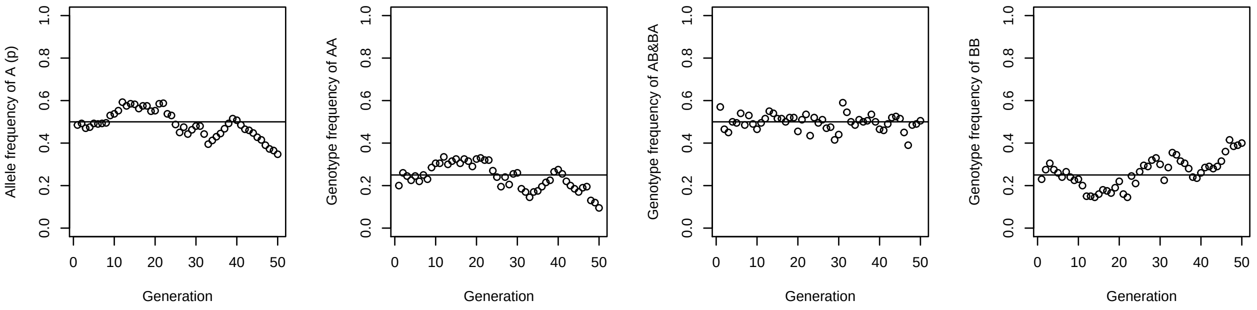

Calculation of fixation indices (F_{IS}, F_{ST}, F_{IT})

Calculate the observed heterozygosity (H_I) based on genotype frequency p_{AA} and the expected heterozygosity (H_S) based on the allele frequency within each sub-population in each generation.

$$H_I = p_{AB}$$
$$H_S = 2p(1-p)$$

```
Hstab <- 2*ptab*(1-ptab)
Hitab <- ABtab
max(Hstab)
```

```
## [1] 0.5
```

Inspect the change of Hs and Hi at two random sub-populations

```
par(mfrow=c(1,4))
for(i in sample(1:r,2)){
  plot(1:nrow(Hitab),Hitab[,i],ylim=c(0.0,1),ylab="Hi",xlab="Generation",main=paste("Hi of sub-populati
  plot(1:nrow(Hstab),Hstab[,i],ylim=c(0,1),ylab="Hs",xlab="Generation",main=paste("Hs of sub-population
}
```

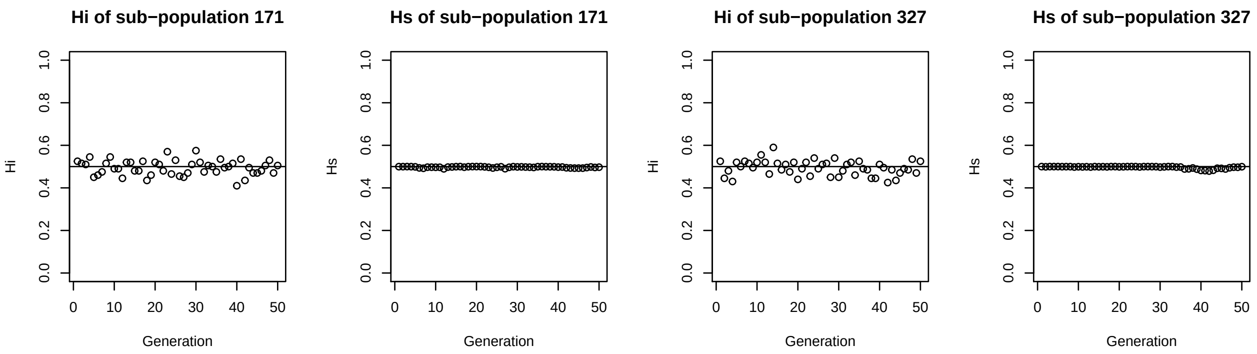

As expected, H_I and H_S is maximum (0.5) at the beginning and decreases over time. Observed heterozygosity (H_I) tends to vary more.

In order to calculate pairwise F_{ST}, n_{pairs} pairs of random sub-populations are drawn and the expected heterozygosity of the combined sub-populations, H_t is calculated for each generation, and F_{ST} is calculated with

$$F_{IS} = \frac{H_S - H_I}{H_S}$$
$$F_{ST} = \frac{H_T - H_S}{H_T}$$
$$F_{IT} = \frac{H_T - H_I}{H_T}$$

```
Fispairtab <- data.frame()  # tabs will have dimension: rows=generations, columns = npairs
Fstpairtab <- data.frame()
Fitpairtab <- data.frame()
npairs <- 1000

for (pairi in 1:npairs){
  sub <- sample(1:r,2) # no replacement to avoid sampling of same population twice
    # average Hi of both populations
    Hi <- rowMeans(cbind(Hitab[,sub[1]] ,Hitab[,sub[2]]),na.rm=T)
```

```
    # Hs: average of within pop exp het (calculated above)
    Hs <- rowMeans(cbind(Hstab[,sub[1]],Hstab[,sub[2]]),na.rm=T)
    # Ht: overall exp het: first average allelefreq and then calculate exp het
    avP <- rowMeans(cbind(ptab[,sub[1]] ,ptab[,sub[2]]),na.rm=T)
    Ht <- 2*(1-avP)*avP
    Fis <- (Hs-Hi)/Hs
    Fst <- (Ht-Hs)/Ht
    Fit <- (Ht-Hi)/Ht

    # if allele frequencies reach same fixation (p=0 in both or p=1 in both), avP will be 0 or 1, Ht an
    Fis[is.na(Fis)] <- 0
    Fst[is.na(Fst)] <- 0
    Fit[is.na(Fit)] <- 0

  # collect
    Fispairtab[1:t,pairi] <- Fis
    Fstpairtab[1:t,pairi] <- Fst
    Fitpairtab[1:t,pairi] <- Fit
  # add population number as column name
    names(Fispairtab)[pairi] <- paste(sub,collapse="vs")
    names(Fstpairtab)[pairi] <- paste(sub,collapse="vs")
    names(Fitpairtab)[pairi] <- paste(sub,collapse="vs")
}
```

Calculate the mean change of the three fixation indices over all pairs of sub-populations.

```
Fismean <- apply(Fispairtab,1,function(x)mean(x))#,na.rm=T)) # sometimes, NAs were produced
Fstmean <- apply(Fstpairtab,1,function(x)mean(x))#,na.rm=T)) # sometimes, NAs were produced
Fitmean <- apply(Fitpairtab,1,function(x)mean(x))#,na.rm=T)) # sometimes, NAs were produced
```

Plot change over generations

```
par(mfrow=c(1,3))
plot(1:t,Fismean,ylim=c(-1,0.5),xlab="Generation",ylab="Mean Fis",main="Fis")
plot(1:t,Fstmean,ylim=c(0,0.2),xlab="Generation",ylab="Mean Fst",main="Fst")
plot(1:t,Fitmean,ylim=c(0,0.2),xlab="Generation",ylab="Mean Fit",main="Fit")
```

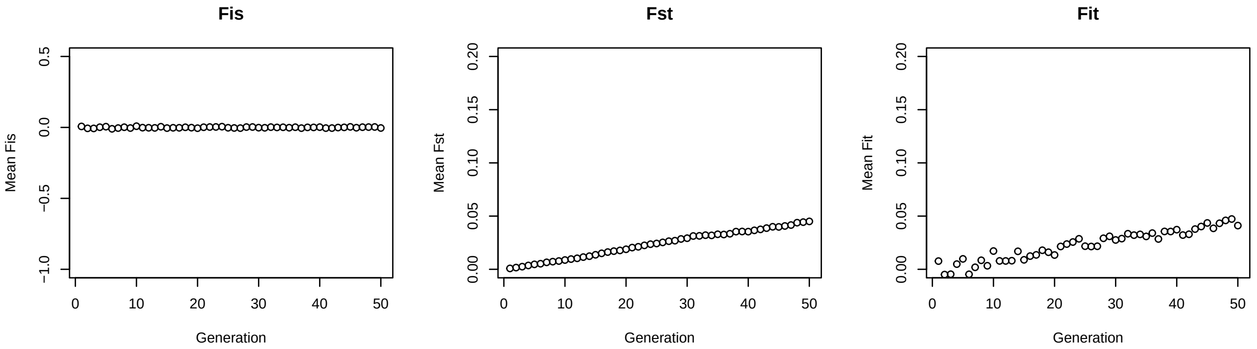

Due to absence of inbreeding, i.e. Hardy-Weinberg equilibrium is maintained within sub-populations, F_{IS} does not increase, the increase in F_{ST} is due to increase in F_{IT}, or vice versa.

Mathematical calculation of F_{ST}

In Hedrick's Genetics of population (p 502) the following equation for the change of F_{ST} due to genetic drift is given (apparently taken from Wright(1943)). Here, N refers to the effective population size of the sup-populations.

$$F_{st} = 1 - e^{-t/2N}$$

```
Fstmath <- 1-exp(-1*(1:t)/(2*Ne))
```

Compare to observed change in F_{ST}

```
plot(1:t,Fstmean,ylim=c(0,0.2),xlab="Generation",ylab="Mean Fst")
lines(1:t,Fstmath,col="red")
```

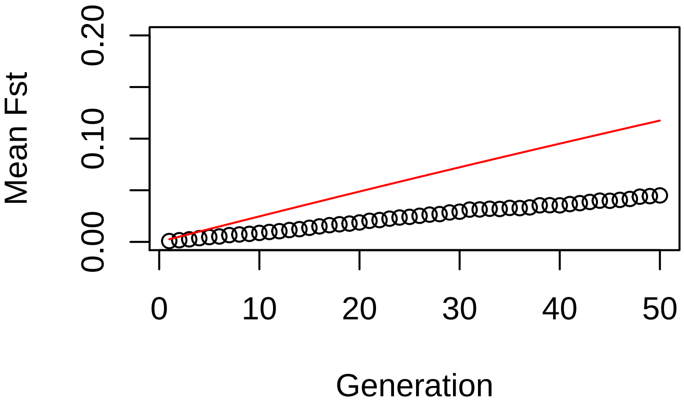

Another equation is given in Hartl and Clark's *Principles of population genetics* (p 117). Although this is actually meant for inbreeding coefficient, it should also apply to F_{ST}

$$F_t = 1 - (1 - (1/(2N)))^t$$

```
Ftmath <- 1-(1-(1/(2*Ne)))^(1:t)
plot(1:t,Fstmean,ylim=c(0,0.2),xlab="Generation",ylab="Mean Fst")
lines(1:t,Ftmath,col="blue")
```

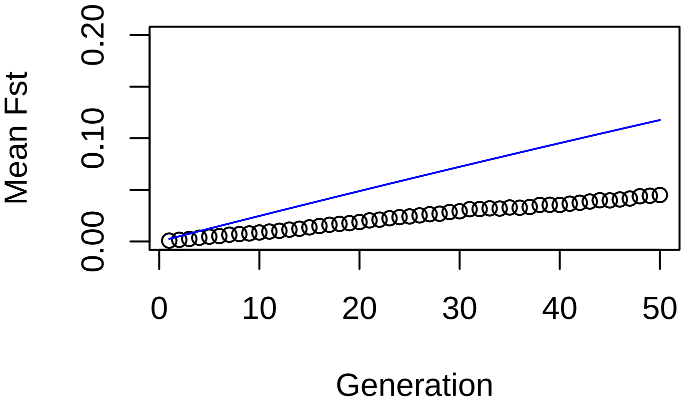

Both equations show the same increase. But don't match the observed values from simulation. The difference could be due to the fact, that the equations measure the deviation from a non-changing or population, or the difference to original population. In the simulation, and in the actual investigation, F_{ST} is measured between pairs of populations.

When using $4N_e$ instead of $2N_e$, the calculated values match the simulated values.

```
Fstmath <- 1-exp(-1*(1:t)/(4*Ne))
Ftmath <- 1-(1-(1/(4*Ne)))^(1:t)

plot(1:t,Fstmean,ylim=c(0,0.2),xlab="Generation",ylab="Mean Fst")
lines(1:t,Fstmath,col="red")
lines(1:t,Ftmath,col="blue")
```

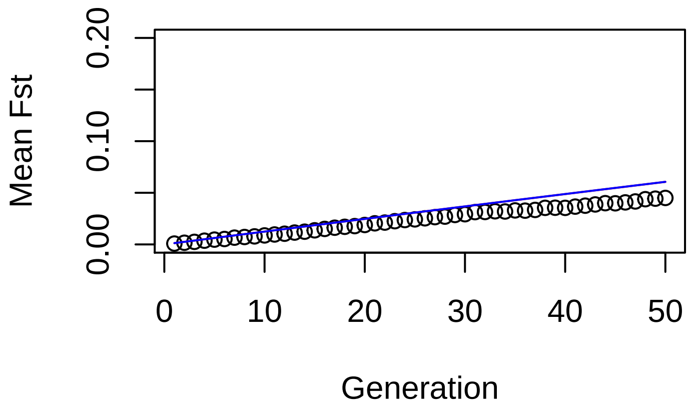

The calculated values match the simulated values of F_{ST} for early generations. As the actual investigation is up to generation, estimates are judged to sufficiently accurate.
